# Targeted Application of Functional Foods as Immune Fitness Boosters in the Defense against Viral Infection

**DOI:** 10.3390/nu15153371

**Published:** 2023-07-28

**Authors:** Dearbhla Finnegan, Restituto Tocmo, Christine Loscher

**Affiliations:** School of Biotechnology, Dublin City University, D09 DX63 Dublin, Ireland; dearbhla.finnegan7@mail.dcu.ie (D.F.); restituto.tocmo@dcu.ie (R.T.)

**Keywords:** functional food, viral immunity, COVID-19, immune fitness, health benefits, elderly, obese, chronic disease, boosting immune system, fermentates, milk hydrolysates

## Abstract

In recent times, the emergence of viral infections, including the SARS-CoV-2 virus, the monkeypox virus, and, most recently, the Langya virus, has highlighted the devastating effects of viral infection on human life. There has been significant progress in the development of efficacious vaccines for the prevention and control of viruses; however, the high rates of viral mutation and transmission necessitate the need for novel methods of control, management, and prevention. In recent years, there has been a shift in public awareness on health and wellbeing, with consumers making significant dietary changes to improve their immunity and overall health. This rising health awareness is driving a global increase in the consumption of functional foods. This review delves into the benefits of functional foods as potential natural means to modulate the host immune system to enhance defense against viral infections. We provide an overview of the functional food market in Europe and discuss the benefits of enhancing immune fitness in high-risk groups, including the elderly, those with obesity, and people with underlying chronic conditions. We also discuss the immunomodulatory mechanisms of key functional foods, including dairy proteins and hydrolysates, plant-based functional foods, fermentates, and foods enriched with vitamin D, zinc, and selenium. Our findings reveal four key immunity boosting mechanisms by functional foods, including inhibition of viral proliferation and binding to host cells, modulation of the innate immune response in macrophages and dendritic cells, enhancement of specific immune responses in T cells and B cells, and promotion of the intestinal barrier function. Overall, this review demonstrates that diet-derived nutrients and functional foods show immense potential to boost viral immunity in high-risk individuals and can be an important approach to improving overall immune health.

## 1. Introduction

In recent years, as a result of the increase in awareness of the impact of diet on health, there has been a significant shift in the interest of consumers towards food that improves immune health. Food is a critical influencer of a healthy, disease-free, high-quality life. Given that global dietary risk factors are estimated to cause 11 million deaths and 255 million disability-adjusted life years annually [1], food has become fundamental to longevity more than ever. It is a long-known fact that what we eat influences our body, and vital nutrients are essential for growth, cellular function, tissue development, energy, and immune defense [2]. There is growing evidence that food can act as an immunomodulator, and certain nutrients and foods have been highlighted to improve immune defense and to increase resistance to infection while maintaining immune tolerance [3]. Furthermore, deficiency in certain nutrients leads to malnutrition and results in the development of diseases. Often, these diseases result from nutritional inadequacies, which impair immune function.

A poor diet can compromise the immune system, leaving the host more susceptible to pathogenic infection, including viral infections. With the recent viral outbreaks, including the SARS-CoV-2 virus, the monkeypox virus, and, most recently, the Langya virus, boosting the immune system is increasingly important. As seen with the COVID-19 pandemic, viruses have the capability of spreading rapidly from person to person, causing devastating damage to humanity due to their potential for high transmissibility. Therefore, there is an urgent need to explore new ways of enhancing viral immunity. Food has long been known to provide antiviral protection and, therefore, can be used as a first-line strategy to boost the immune system in the form of functional foods to enhance protection against new, emerging viral infections as well as well-established viruses, including the influenza virus [4,5,6,7,8,9,10,11].

Used in conjunction with hygiene practices and an active lifestyle, functional foods may provide additional, naturally sourced antiviral protection. In this review, we discuss the current trends in health and wellbeing in relation to the growing interest in functional foods, especially in Europe. We present the concept of boosting immune fitness with a focus on individuals at high risk of viral infections. Moreover, we discuss the mechanisms by which diet-derived nutrients and functional foods can boost immunity in high-risk individuals, and how diet-based strategies can be an important approach to improving overall immune health. Finally, we present future perspectives on how functional foods, especially fermented foods, can be further developed for boosting immune fitness.

## 2. Trends in Health and Wellbeing: A Focus on Functional Foods

The increased popularity and global explosion within the Health and Wellbeing industry are evidenced by the booming economic figures in data from 2015 to 2017, showing a growth rate of 6.4% [12]. This growth was expected to continue at this pace, and the global wellness industry had a net worth of an estimated USD 4.75 trillion in 2019 [12]. The total Healthy Eating, Nutrition, and Weight Loss sector comes to an estimated USD 702 billion, making it the second largest sector of the industry [12].

**Figure 1:**
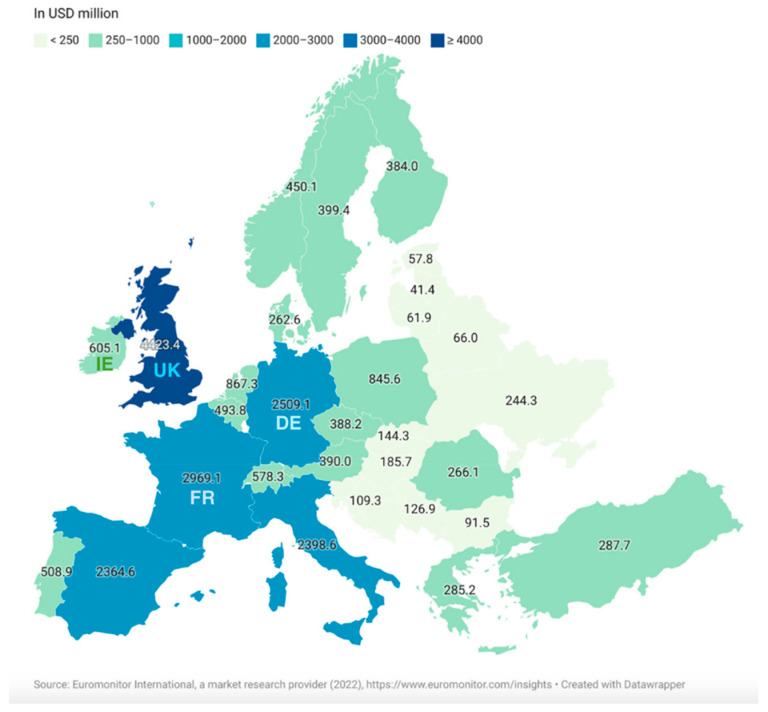
Retail sales value of functional foods in Europe in 2021 [13]. Two-letter country abbreviations included for markets of interest. Adapted from [14].

**Figure 2:**
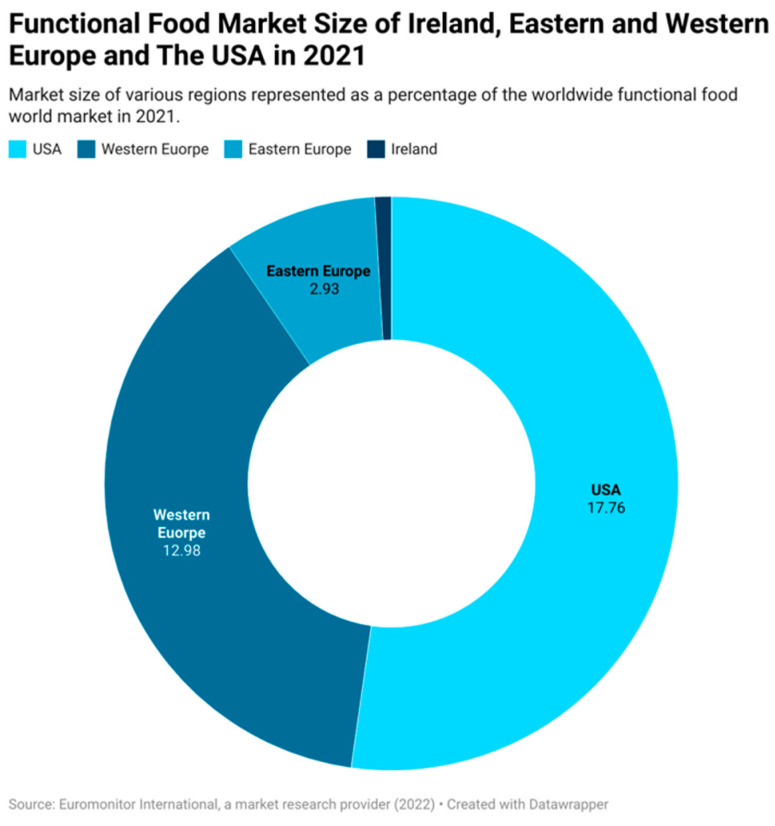
Percentage hold of various regions of the worldwide functional food market size for 2021. Data obtained from Euromonitor International, a market research provider (2022) [13]. Created with Datawrapper.

These trends in the Health and Wellbeing industry reflect trends seen in the food industry, with an increasing demand for healthy foods, in turn driving the revolution that is the development of food products that can impart additional health benefits to their consumers, i.e., the functional food industry. The functional food industry is growing at a phenomenal rate, with a worldwide growth rate of 10.34% from 2016 to 2021 based on data gathered from Euromonitor [13]. Furthermore, this growth rate is expected to increase further, nearly doubling that of 2016–2021, with an expected worldwide growth rate of 19.17% from 2021 to 2026 [13]. The retail sales value of the functional food industry in Europe in 2021 was dominated by the UK, which held the largest value, followed by France and Germany (Figure 1) [14]. The popularity of functional foods worldwide is on the rise, with the USA, Europe, and Japan being the regions holding the greatest retail sales values [14]. An annual increase in market size for Ireland, Eastern and Western Europe, the USA, and worldwide has been observed (Table 1) with future predictions in market size for 2022–2026. Figure 2 shows the percentage hold Ireland, Eastern and Western Europe, and the USA have in the worldwide functional food market, with Europe and the USA making up over a third of the worldwide functional food market at a combined 33.67% of the total market size.

This annual growth rate was expected to continue for the foreseeable future; however, in late 2019 and early 2020, the catastrophic news of a global COVID-19 pandemic further ignited global interest in the Health and Wellness industry. The rapid growth and boom within the functional food industry are clear evidence that, globally, we are now looking to food as a source of immune support when food is so well known to aid in anti-viral immunity and overall immune support [4,5,6,7,8,9,10,11]. Seeking functional food sources with immunomodulatory capabilities could potentially be a game changer to naturally aid our management of viral infection.

Due to the current economic climate and a recent global pandemic, the immune support and health supplements market is expected to grow even further, at a compound annual growth rate of over 9% from 2019 to 2025 [15]. With an estimated six-month immunity provided by the vaccines as antiviral therapy for the prevention of COVID-19 infection, alternative methods to augment protection against viral invasion are highly desirable [16,17].

## 3. Boosting Immune Fitness in High-Risk Individuals

In this section, we highlight the importance of boosting the immunity of high-risk individuals. We first present the concept of immune fitness, followed by an overview of the key features of the innate and adaptive immune system of three highly vulnerable cohorts with a focus on gut immunity. Understanding the changes in the immune responses of these cohorts is critical to furthering the development of functional foods targeted at boosting viral immunity.

### 3.1. Immune Fitness

**Figure 3:**
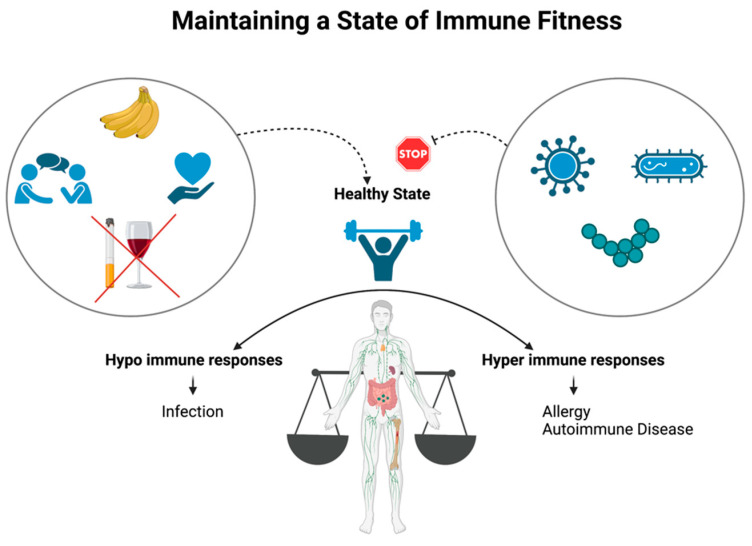
Maintaining immune fitness. Good diet, social relationships, stress control, and not smoking or drinking all aid in maintaining a healthy immune system to enable the immune system to fight off pathogens and recognise harmless food antigens. The ability and resilience of the immune system to fight off and manage such challenges are encapsulated by the immune fitness of the individual. Hypo immune responses lead to the development of infections. Hyper immune responses lead to the development of allergy and autoimmune disease. Created with BioRender.com.

Immune fitness describes the capacity of the body to respond to health challenges, such as infection, via activation of the appropriate immune response in order to prompt disease resolution, prevent pathogen infection, and promote health, thereby ensuring quality of life [18]. Immune fitness refers to a resilient immune system with the “built-in” capacity to adapt to challenges by establishing, maintaining, and regulating an appropriate immune response [19]. This means that the individual’s immune system is robust enough to eliminate harmful pathogens, such as viruses and bacteria, while simultaneously being able to tolerate harmless ones, such as food antigens. In doing so, this prevents the body from entering into a hyporesponsive state of weakened immunity, leading to increased infection, and from entering into a hyperresponsive state, leading to allergy and autoimmune disease (Figure 3) [19]. Immune fitness can be viewed as the establishment of core lifestyle habits that can improve your immune capacity, including good eating habits, good social relationships, abstinence from smoking, limiting alcohol consumption, and controlling stress levels, all of which can slow down the process of aging on the immune system [20].

As we age, the immune system changes, and chronic low-grade inflammation develops, termed “inflammageing”, which, in turn, contributes to the pathogenesis of age-related disease [21]. The immune system responds more slowly and less effectively; thus, there is increased risk of infection due to less effective immune defenses. This gradual deterioration in the immune system caused by advanced aging is termed immunosenescence [20]. Nutrition is closely linked to proper functioning of the immune system, meaning what we eat has a huge influence over our immune response, making us more or less likely to suffer from infections or inflammatory disease [22]. It is long understood that the plant-based Mediterranean diet, consisting largely of cereals, legumes, vegetables, fruits, olive oil, and nuts, provides fibre with prebiotic activity, polyunsaturated fatty acids with anti-inflammatory properties, bioactive compounds with antioxidative properties, and vitamins and minerals, all aiding in the modulation of the microbiota, the activation of the host immunity, and, ultimately, promoting health and disease prevention [23]. On the other hand, the Western diet is associated with high animal protein, digestible sugars, and fat, while also being low in fibre [24]. Observational studies have linked the Western diet to the risk and development of inflammatory bowel diseases (IBDs), including Crohn’s Disease (CD) and ulcerative colitis (UC), as well as other immune diseases, including asthma and allergy, while also coinciding with an increase in autoimmune diseases [25,26,27,28]. Furthermore, the Western diet is a key contributor to the global obesity epidemic, which causes low-grade activation of the immune system [28]. Therefore, the differing effects of the Mediterranean and Western diets demonstrate that differing dietary patterns have differential effects on the immune system and overall immune fitness. Individuals who are considered immunocompromised are those with weakened immune systems [29]. These people have a reduced ability to fight infections and other diseases. Certain conditions, like cancer, diabetes, AIDS, some genetic conditions, and even simply malnutrition, result in an individual becoming immunocompromised [30]. Not only this; often, the treatments for various diseases like cancer, including radiation therapy or stem cell therapy, result in immunosuppression [29]. The immunocompromised are a highly vulnerable group with reduced immune system response and thus require boosting of their immune fitness in order to help tackle any immune challenges they may face.

In this review, we examine the effect of functional foods on the immune fitness of three core vulnerable populations within our society: the elderly, the obese, and the immunocompromised. Challenges arise in the fight against viruses, such as COVID-19, in the elderly population, obese individuals, and for those who suffer from chronic underlying conditions, as these individuals have an already weakened immune system. Individuals that are older and have underlying conditions are at an increased risk of severe infection due to their already weakened immune systems [31]. It is the low-grade inflammation within the immune systems of the elderly and the obese that makes them vulnerable to infection [32,33]. The aging process is inevitable; however, there are other factors one can consider to keep the body as fit and healthy as possible to manage weight and to support the immune system in order to protect against viral invasion. Immune fitness encapsulates how one’s immune system is built in terms of its resilience, fragility, and chronic immune disorder morbidity [34]. Immune fitness can be influenced by a variety of factors, including biological factors, such as the epigenome and microbiome, lifestyle factors, such as sleep, diet, and exercise, and other psychosocial factors, like stress response and the social environment [34]. Therefore, it is critical that we look for natural ways of boosting the immune fitness of the elderly, the obese, and the immunocompromised to ensure the immune system is in a prime state for fighting against viral infection by virtue of maintaining a healthy lifestyle.

#### 3.1.1. Immune Response in the Elderly

The elderly are classed in the extremely vulnerable group of individuals at risk of contracting viral infections, including COVID-19 infection, while people less than 65 years old have been shown to have a smaller risk of COVID-19 deaths, even in pandemic epicentres [35]. Not only are the elderly at increased risk of susceptibility to infectious disease, but they are also seen to have reduced vaccine efficacy [36]. As we age, there is an increased dysregulation of the immune system, both innate and adaptive, due to the accumulation of damage over the years at a molecular, cellular, and organ-based level, which results in the increased risk of disease and higher rates of morbidity and mortality [37]. The degree to which the immune system is affected by the aging process is referred to as immunosenescence [38]. The degree of immunosenescence can be slowed down by optimising the immune fitness of the individual [39].

As we age, the innate immune system is highly affected. The mucosal immune system of the gastrointestinal tract, which provides the first line of defense against ingested pathogens by deciphering between harmless antigens and generating tolerance towards them or being able to mount a rapid protective immune response against dangerous pathogens, becomes significantly compromised in the elderly [40]. The intestinal mucosa provides innate immunity by virtue of the gut-associated lymphoid tissue (GALT), constituted by Peyer’s patches, lymphoid follicles, and mesenteric lymph nodes [41]. In aged GALT, reductions in mucus secretion and defensins are reported, indicative of impaired gut function [42]. Aging brings about an increase in the innate cells of the intestine with an elevation of pro-inflammatory mediators, i.e., pro-inflammatory cytokines (IL-6, TNF-α, IFN-γ, and IL-1β) and C-reactive protein, contributing to the low-grade inflammation associated with aging, as well as a link to poorer cognitive performance [43]. Aged dendritic cells show significant decreases in IL-12p70 and IL-15 production and decreased expression of co-stimulatory factors CD80/CD86 [44,45], affecting their ability to present antigens that activate cells of the adaptive immune system. Furthermore, a link to reduced expression of IL-22 and IL-17 cytokines results in increased intestinal barrier permeability in the elderly [46]. Furthermore, an increase in IL-6 and TNF-α is associated with the increase in paracellular permeability of the microbiome as well as metabolic endotoxemia, which contributes to the low-grade chronic inflammatory state seen in aging [47]. Changes in the gut microbiome are often associated with aging, where a decrease in microbiota diversity is observed, and an increase in pro-inflammatory commensals is seen at the expense of beneficial microbes [48]. Such dysbiosis leads to alterations in microbiota-associated metabolite levels, impaired function and integrity of the gastrointestinal tract, and increased leaky gut [48]. In addition, reductions in chewing ability, dentition, taste, digestion, and intestinal transit time affect dietary choices and food digestion as we age, contributing directly or indirectly to microbiota alterations [49]. Therefore, as we age, the integrity and functioning of the gut are compromised, leading to increased permeability of the gut by virtue of its decreased integrity, meaning the elderly are at increased risk of viral infection due to this weakened first line of physical defense.

The adaptive arm of the immune system is highly affected by aging, with some key changes seen, such as decreased T cell function, the central defect of immunosenescence, as well as decreased production of T cell populations. Aging is associated with declined IL-2 production and expression of T cell receptors, resulting in decreased T cell proliferation, as well as altered signaling pathways associated with T cell activation, i.e., the NF-κB pathway [37,50]. T cell function is therefore already impaired in an older individual; thus, contracting COVID-19 and the associated T cell exhaustion (Tex) puts these individuals at a much greater risk of a poor outcome. Similarly, B cell function is affected by the aging process, making the elderly defective in their ability to produce optimal antibody responses [51]. These defects include reduced somatic hypermutation of the antibody variable region, reduced binding, reduced class switch recombination responsible for the generation of a secondary response of class switched antibodies, reduced neutralization capacity and binding specificity of secreted antibodies, increased frequencies of inflammatory B cell subsets and shorter telomers, and increased epigenetic modifications that are associated with lower antibody responses [51]. Furthermore, there is an obvious effect seen on B cell function in an aged immune system. The number of competent B cells significantly decreases with age, while the percentage of terminally differentiated and senescent memory CD27^-^ B cells increases [52]. Thus, the elderly have an impaired antibody function, which only further serves as a potential risk factor for contracting viral pathogens.

The gut undergoes several changes in the adaptive immune response as a result of aging. On a cellular level, the ability of dendritic cells to initiate T cell responses is impaired due to defective priming by dendritic cells of the mucosal tissue, while antigen-specific T cell responses in the gut are reduced [44]. The expression of co-inhibitory molecules CTLA-4 and PD-1 in lamina propria CD4^+^ T cells, which control homeostasis and antigen-specific responses, is significantly lower in the elderly [53,54,55]. Furthermore, older Th1 and Th17 cells proliferate to a lesser degree compared to their younger T subset counterparts, while higher levels of spontaneous death among older CD4^+^ T cells are observed [54].

Immunoglobulin A (IgA) is the dominant class of antibody secreted by the intestinal mucosa, and it plays a key role in the regulation of the gut microbiota [56]. IgA is a major class of antibody secreted by the gut mucosa and is key to the maintenance of intestinal homeostasis [57], gut immunity, regulating the mucosal immune response, maintaining the microbiome [58], and activating the gut microbiome to promote protection from inflammation [59]. T cells regulate the magnitude and nature of microbiota-specific IgA via IgA-committed B cell responses [40]. However, these become senescent as we age, and it is therefore suggested to be a contributing factor in the decreased antigen-specific IgA responses associated with aging [56]. This reduction in IgA response is, in turn, thought to contribute to the decline in gut and intestinal immunity as we age. Specifically, this reduction in IgA is linked to the decreased small intestinal CCL25 and the increase in colonic CCL28 associated with advanced aging and the deterioration of gut immunity [58].

Overall, the elderly have a unique pro-inflammatory predisposition. They experience a constant low-grade inflammation (LGI) that leads to the chronic systemic inflammation that is strongly associated with the elderly population and is the causative factor of many age-related illnesses, such as cardiovascular disease and stroke, and autoimmune disorders, such rheumatoid arthritis [60]. Thus, in the context of viral infection, such as COVID-19, an elderly individual will already have a heightened inflammatory response and is at a significantly higher risk of having a more severe outcome from viral infection.

#### 3.1.2. Immune Response in Obese Individuals

Obesity is an excessive accumulation of adipose tissue, clinically defined as constituting a body mass index (BMI) > 30 kg/m^2^ [61]. Obesity is linked to a reduced oxygen saturation of the blood by compromised ventilation in the base of the lungs [62]. It is characterised by chronic low-grade systemic inflammation, with an increased pro-inflammatory cytokine profile in adipose tissue and infiltration of leukocytes, such as macrophages, into the adipose tissue [63]. Such chronic inflammation results in impaired insulin signaling in adipocytes, causing insulin resistance and further contributing to the development of metabolic disorders, such as cardiovascular disease, type 2 diabetes, and hypertension [64].

Obesity has long been known to be linked to an increase in susceptibility to viral infections, including influenza A virus and swine flu [65]. Comorbidities linked to obesity can result in an increased risk of worse prognosis for COVID-19 and may even require mechanical breathing [66]. Obese individuals are more likely to suffer from other independent risk factors for severe COVID-19 than normal-weight-bearing individuals, including heart disease, lung disease, and diabetes [67] due to their added weight, poorer diet, and reduced exercise compromising their metabolic health. Even when vaccinated, obese adults tend to contract viral infections more easily than healthy weight individuals. For example, obese individuals are twice as likely to develop influenza or influenza-like illness compared with healthy weight adults post influenza vaccination [68]. Therefore, the risk of contracting COVID-19 and other viral infections in obese individuals is higher because they already have decreased lung capacity and difficulty breathing, as well as chronic low-grade inflammation. This risk of contracting a viral infection puts them at a greater risk of increased susceptibility, poorer prognosis, increased severity of disease, and increased mortality rates.

Innate immunity is highly affected in the gut of obese individuals, making them more vulnerable to viral diseases. Macrophages are key players in innate immunity. They are major mediators of inflammation within adipose tissues and are the most abundant immune cells that contribute to obesity via the infiltration of adipose tissue and subsequent secretion of inflammatory cytokines in response to obesity [69,70]. A lean profile is associated with the “alternatively activated” M2 macrophage phenotype, while an obese profile is associated with the “classically activated” M1 macrophage phenotype [69]. It is the breach of the intestinal barrier that induces microbe-associated molecular patterns to stimulate intestinal epithelial cells (IECs) and macrophage, and dendritic cells to produce proinflammatory cytokines, such as IL-1, IL-6, IL-12, IL-18, and IL-23, which result in the intestinal cytokine profile associated with diet-induced obesity and often resulting in insulin resistance [71,72]. Intestinal permeability is increased as a result of obesity by virtue of a high-fat diet that sparks an imbalance in the gut microbiota diversity and alters the microbial composition [71,73]. This imbalance initiates an innate immune response triggered when pathogens cross the intestinal barrier with greater ease [71,73]. This increased intestinal permeability is due to the reduced expression of epithelial tight junction proteins, such as zonula occludens 1 (ZO-1) and claudins [74]. IFN-γ-secreting immune cells, i.e., M1 macrophages [69], are in part responsible for barrier permeability, as IFN-γ reduces ZO-1 expression in intestinal epithelial cells [75]. Similarly, IL-1β has been linked to the increase in intestinal epithelial tight junction permeability [76]. Macrophages are recruited due to the secretion of chemokines and the activation of pattern-recognition receptors (PRRs), such as toll-like receptors (TLRs), that recognise unique danger signals for the differentiation of pathogens for the neutralization of pathogens or clearing of stressed cells induced by obesity [69].

Obese adipose tissues have been noted to have increased macrophage accumulation and higher TNF-α and IL-6 cytokine levels; thus, obesity is associated with an accumulation of immune cells that, overall, contribute to a state of LGI, dysregulated metabolism, and insulin resistance as a result of this pro-inflammatory state [77]. Furthermore, the bone marrow, which is the site where immune cells develop, is affected by obesity in that ectopic fat accumulates here, thus affecting immune cell development [78]. Circulating PBMCs have been shown to secrete higher levels of TNF-α and lower levels of the anti-inflammatory cytokine IL-10 in obese individuals, which further establishes this permanent state of chronic inflammation [79]. In addition, TLR activation of PBMCs becomes impaired in obese people, with a decreased ability to produce antiviral type I IFNs, IFN-α, and IFN-β [80]. The chemoattractant monocyte chemoattractant protein-1 (MCP-1) is secreted by adipose tissue and macrophages and is more abundant in obese individuals than in lean individuals [81]. MCP-1 is stimulated by the presence of IL-1β, TNF-α, IL-8, IL-4, and IL-6, and thus further aids in the macrophage recruitment to adipose tissue seen in obesity [81].

It has been proven that obesity-related disease progression and severity are highly correlated with pro-inflammatory T and B cell phenotypes within the gut [69]. Gut resident T cells include CD8^+^ T cells and CD4^+^ T cells consisting of Th1 cells, Th2 cells, Th17 cells, Tfh cells, and Treg cells [82]. It is the CD4^+^ Th17 and Treg cells that are most abundant in mucosal tissue [83]. Normal lean homeostatic conditions within the intestinal immune environment lead to immune cells, which are dominated by tolerogenic and mucosal-barrier-maintaining cells, such as IL-10-producing Treg cells, IL-22-producing lymphoid cells, and IL-17-protective Th17 cells, as well as IgA^+^ antibody-secreting cells producing secretory IgA to interface within the lamina propria [84]. Diet-induced obesity in mice demonstrates a shift in the inflammatory response within the intestinal immune environment, which leads to increases in Th1 and CD8^+^ T cells, a decrease in the aforementioned tolerogenic cell types Th17 and Treg [83], a decrease in intestinal homing CCR2^+^ macrophage, a decrease in intestinal intra-epithelial CD8αβ^+^ T cells, and an increase in IL-2, via small intestinal group 2 innate lymphoid cells. These all promote diet-induced obesity via intestinal dysfunction that enables dysregulated glucose homeostasis [84]. Obesity is associated with fewer intestinal IgA^+^ immune cells and secreting less secretory IgA and IgA-promoting immune mediators, which results in dysfunctional glucose metabolism of the microbiome [85]. IgA is a critical B-cell-induced antibody that controls intestinal and adipose tissue inflammation, intestinal permeability, microbial encroachment, and the composition of the intestinal microbiome [85]. Limiting IgA secretion greatly impacts the gut immune system, further showing the detrimental effects associated with obesity on the gut. IgA is essential for gut homeostasis, and reduced levels demonstrated in obesity results in altered gut microbiota and further suggests a crucial supporting role for intestinal immunity as a key modulator of the systemic glucose metabolism microbiome [85].

The increased Th1 and Th17 subpopulations in obese individuals further contribute to the heightened proinflammatory state, which is detrimental to the body when under viral attack. Therefore, obese individuals are already in a state of LGI, cytokine dysregulation and T cell pro-inflammatory activation, putting them at higher risk of poorer prognosis in the event of contracting viral diseases, including COVID-19.

#### 3.1.3. Immune Response in People with Underlying Chronic Conditions

People with underlying health conditions are at serious risk of contracting viral infections that may result in the need for hospitalisation, intensive care, ventilators, or mechanical machinery to help them survive, as they are at a higher risk of death due to the severity of the illness [86]. The weakened immune system puts these individuals at great risk for contracting viral infections as they have medical conditions and/or are undergoing treatments for medical conditions that suppress their immune system. Examples of immunocompromised cohorts include individuals suffering from CD or UC, HIV patients, cancer patients, and organ transplant recipients whose underlying conditions only serve to amplify the effects of COVID-19 [86]. In this section, we focus our discussion on the key features of the gut immunity in people living with HIV and those with IBD.

HIV is a virus that attacks the individual’s immune system and weakens its ability to fight infection and disease, thus putting the HIV-positive individual at a higher risk of contracting other viral infections and suffering from poorer clinical outcomes. HIV damages the immune system by infecting CD4 cells that help fight off infection and protect the body from disease [87]. HIV affects approximately 37.7 million people worldwide, while 680,000 people died of HIV-related illness worldwide in 2020 [88,89], with no known cure [90].

HIV infection alters the components of the gut microbiome and changes the host immune responses to gut microbes, which means the gut plays a critical role in the immune systems of HIV-positive individuals [91]. HIV is associated with a chronic inflammatory state represented by increased soluble IL-6 and high-sensitivity CRP, D-dimer, and cystatin C levels even after antiretroviral therapy [92]. The gut epithelial barrier integrity, including intestinal fatty acid binding protein and zonulin-1 levels, as well as innate immune activation and inflammation through markers like soluble CD14 levels, kynurenine/tryptophan ratio, and TNF receptor 1 levels, are highly affected in HIV infection, with increased levels being strong independent predictors of mortality [93]. The gradual loss of CD4 T cells in HIV has a knock-on effect for the innate immunity provided by the gut, as the poor CD4 cell recovery within the lamina propria results in the disruption of the gut mucosal barrier integrity. Therefore, the first line of innate defense against invading pathogens becomes weakened, and there is a subsequent loss in cytokines secreted, which are needed for the support of normal barrier function [94].

The leaky gut barrier leads to systemic inflammation due to increased circulation of microbial components in the bloodstream as well as an increase in exposure of the resident gut mucosal T cell population to new antigens, meaning the gut barrier dysfunction seen with HIV infection may originate in the gut lamina propria and its resident CD4 T cells [91]. Lamina propria T cells are thought to be more susceptible to HIV infection due to the high levels of activation and expression of HIV receptors like CCR5 [95]. HIV infection is associated with the gradual loss of peripheral CD4^+^ T cells, largely through the accelerated proliferation, expansion, and death of T cells, and this high T cell turnover results in the depletion and exhaustion of the regenerative capacity of the hyperactive immune system, leading to opportunistic infections, malignancies, and, ultimately, death [96]. It is this exhaustion of the immune system that leads to the subsequent development of acquired immunodeficiency syndrome (AIDS), whereby the immune system is unable to maintain the high rate of T cell production it requires for proper functioning [96]. This hyperactivation of the immune system on naïve T cells, whether antigen-specific, induced by cytokines, or by viral gene products, may lead to the increased consumption of both CD4^+^ and CD8^+^ naïve T cells through apoptosis of activated T cells or differentiation towards memory phenotypes [96,97,98]. Decreases in sigmoid IL-22-producing CD4^+^ T cells, which are essential for the sigmoid mucosa integrity, are also observed in HIV infection, thereby worsening epithelial barrier dysfunction and increasing microbial translocation [91,94].

IBD is associated with gut discomfort as a result of immunological imbalances within the intestinal mucosa associated with cells of the adaptive immune system [99]. IBDs arise as a result of the immune system responding to self-antigens and triggering chronic inflammation in patients with diseases like UC and CD. The prevalence of IBDs is on the rise [100,101], with these chronic digestive diseases affecting over 10 million people worldwide; they have no known cause or cure [102].

With the modern diet consisting of greater meat and animal product consumption, observational studies have linked such dietary patterns to the risk and development of IBDs like that of CD and UC [27]. IBD is associated with the disruption of the integrity of the epithelial cells of the intestinal lumen bacteria, which are necessary to communicate with the immune system [103]. IECs are key players within the mucosal barrier, preventing the influx of antigens, the invasion of pathogens and commensal microorganisms, and maintaining tolerance to alimentary antigens and commensal microbiota, while also playing a crucial role in the activation of cellular innate and adaptive immune responses, producing cytokines and chemokines, and keeping the epithelial barrier intact [103,104]. Macrophages play a crucial role in the regulation of gut homeostasis within the intestinal mucosa, and when this macrophage function becomes disrupted, this leads to chronic intestinal inflammation [103]. Regulation of intestinal gut inflammation is largely due to the M2 macrophages that produce IL-10 [103]. Dysregulation in IL-10 function leads to a decrease in the secretion of this anti-inflammatory cytokine and is therefore linked to the pathogenesis of IBDs, particularly for UC [105]. Nucleotide-binding oligomerisation domain 2 (NOD2) is a protein encoded by the caspase recruitment domain-containing protein (CARD), which is an intracellular microbial sensor that acts as a potent activator and regulator of inflammation [99]. NOD2 mutation or deficient expression is often associated with IBDs, including CD, through increased expression of inflammatory factors [106]. TLR signaling helps protect the epithelial barrier and aids in tolerance to commensal bacteria; however, a malfunction in the TLR signaling associated with IBDs induces an intestinal inflammatory response through the activation of the NF-κB transcription factor, which regulates the expression of key inflammatory cytokines IL-1, IL-2, IL-6, IL-12, and TNF-*α* [104].

Inflammatory disorders, such as IBDs, are largely associated with issues with the adaptive immune system and consist of alterations in the autophagy of cells, antigen processing, regulation of cell signaling, and T cell homeostasis [99]. The imbalance of Th1 and Th2 cytokine release by the intestinal mucosa determines the development and persistence of inflammatory responses leading to chronic inflammatory disease [107]. Key Th1 cytokines linked to the development of IDBs are TNF-*α* [108], TGF-*β* [109], IFN-*γ* [110], IL-6 [103], IL-12, and IL-18 [111], as well as the response to self-antigens [99]. Similarly, Th17-related cytokines, such as IL-17 and IL-22, play a role in the development and establishment of IBDs [111,112]. Thus, T cells and their subsets may have excessive increases in the chemokines and cytokines that lead to the worsening or maintenance of mucosal inflammation [111]. Treg cells are associated with the pathogenesis of IBD [113]. CD4^+^ and CD25^+^ Treg cells play a role in immune regulation and IBD treatment in mice models, whereby these cells are recruited to the intestinal lymphatic tissues and lamina propria, playing a key role in maintaining intestinal homeostasis [111,114]. Reduction of Treg cells can result in the development of IBD [115]. In patients with IBDs, there is a dysregulation in the amount of antibodies produced and secreted from B cells [116]. UC is characterised by the infiltration of IgG-producing plasma cells via the CD184 receptor of inflamed mucosa, further exacerbating inflammation through the activation of intestinal CD14^+^ macrophages [103,117]. CD is characterised by high levels of IgG1, IgG2, and IgG3 in both serum and intestinal mucosa [118]. Similarly, higher levels of IgM are associated with IBD pathogenesis [119].

Immunocompromised individuals with uncontrolled HIV and those who suffer from IBDs are in a state of chronic T cell exhaustion and chronic low-grade inflammation. These conditions show how highly affected the gut becomes when the body is in a weakened state of immunity and demonstrate the vulnerability of the host to further viral infection. Dietary interventions that enhance immune fitness may benefit people suffering from these conditions.

## 4. Functional Foods as Immune Fitness Boosters in the Context of Viral Infection

The concept of functional foods is thought to have first arisen in Japan less than 40 years ago, with the Japanese initiating the concept of functional food science based on the words of the ancient Chinese, in which they stated that “Medicine and food are isogenic” [120]. This area of food science research gained huge interest and popularity; however, the term “functional food” is still not recognised as a unique regulatory product category by the FDA and has no legal definition [121]. While the area of functional foods is rapidly emerging and has yet to be legally defined in EU or Irish food legislation, it is regulated through existing food legislation instead [122]. There are many global definitions of what a functional food is (Table 2). For the purpose of this review, we define a functional food to be a “Natural or processed foods that contains known or unknown biologically-active compounds; which, in defined, effective, non-toxic amounts, provide a clinically proven and documented health benefit for the prevention, management, or treatment of chronic disease” [123].

A recent review by Zhang et al. [128] noted the importance of vitamins A, B2, B3, B6, C, D, E, omega-3 polyunsaturated fatty acids, selenium, zinc, and iron in the fight against viral infections. These are key traditional functional food components that potentially have the ability to help in the protection against viral infections. In this review, we focus on milk proteins, fermentation products, other plant-derived products, Zinc, selenium, and vitamin D as functional foods with the potential to combat viral infections. In this section, we highlight their interactions with the immune system and the mechanisms underlying their immune-boosting activities.

### 4.1. Whole Milk Proteins and Hydrolysates

There are two groups of proteins in milk: casein and whey. Casein comprises 80% of total bovine milk protein, and the remaining 20% is whey protein. Whey is the major by-product generated from the cheese making industry [129] and is composed of β-lactoglobulin, α-lactalbumin, serum albumin, immunoglobulins, lactoferrin, and transferrin. Casein, on the other hand, is composed of various protein fractions, including αs1, αs2, β-, and κ caseins [130]. Milk-derived proteins can work in a variety of ways to act as antiviral molecules. These traditional antiviral mechanisms include binding to structural viral proteins to prevent host–cell interactions, interfering with viral entry through viral and/or cell surface interaction, as well as by interfering with certain viral enzymes required for viral replication [130]. Most of the antiviral properties attributed to milk are associated with whey proteins, largely lactoferrin; however, casein has also been shown to exert some antiviral activity towards viruses [131]. 

#### 4.1.1. Whey

Most whey proteins have been shown to prevent viral infection [130]. Whey protein from human breastmilk was shown to effectively inhibit both SARS-CoV-2 and its related pangolin coronavirus via blocking viral attachment and viral replication at entry into the cytoplasm and post entry points, as well as by inhibiting infectious viral production [132]. Specifically, whey protein of human breastmilk significantly inhibited the RNA-dependent RNA polymerase (rdRp) activity of SARS-CoV-2 in a dose-dependent manner [132]. This is thought to be due to the rich lactoferrin content, well known for its antimicrobial effects, as well as other components found in breastmilk. Lactoferrin is a naturally occurring nontoxic glycoprotein that has been proven to help protect against viral infections, including SARS-CoV, which is closely related to SARS-CoV-2, which causes COVID-19 [133]. Lactoferrin has demonstrated the ability to inhibit many viruses, including hepatitis B and C viruses (HBV and HCV), herpes simplex viruses 1 and 2, HIV, human cytomegalovirus, human papilloma virus (HPV), enteroviruses, adenoviruses, influenza viruses, parainfluenza viruses, and rotaviruses [134]. For example, it inhibits the activity of reverse transcriptase, protease and integrase, and HIV-1 enzymes, which allow viral replication to occur; thus, lactoferrin can inhibit the viral replication of HIV [134,135].

Lactoferrin has immunomodulatory and anti-inflammatory properties that can be used to confer protection in host systems by modifying host responses to infections through its iron-binding capacity, its direct interaction with cell surfaces, its ability to promote immune cell activation, differentiation, and proliferation, as well as its ability to downregulate immune responses via anti-inflammatory cytokine activity [136]. For example, lactoferrin induces the expression of type I interferon IFN-α/β, known potent antiviral cytokines and immunomodulators, and inhibits viral replication [137]. It has also been shown to lower IL-6 and TNF-α, key players in the cytokine storm [44].

Another potential mechanism is through the inhibition of ACE2 and S glycoprotein. ACE2 is the receptor and main landing site for SARS-CoV-2 on host cells via the spike protein [138]. This spike protein, the S glycoprotein, plays an essential role in virus attachment, fusion, and entry into host cells [139]. Thus, through inhibition of the surface S glycoprotein, ACE2 receptor binding can be prevented, thereby inhibiting viral attachment and subsequent infection. A study by Fan et al. [132] revealed that whey can slightly block the affinity of ACE2 and the S glycoprotein.

In an observational study by Serrano et al. [140], they were able to elucidate a potential dose for the prevention and treatment of COVID-19 infection using liposomal lactoferrin, Lactyferrin™, as follows: a dose of 64–96 mg (20–30 mL) every 6 h daily (256–384 mg/d), which can be increased to 128 mg every 6 h (512 mg) if needed to cure COVID-19, while a dose of 64 mg two to three times daily can prevent COVID-19 (128–192 mg/d). This study allowed for complete and fast recovery of all 75 patients within the first 4–5 days, while smaller doses prevented individuals directly in contact with the patient from ever becoming infected. In another study, low COVID-19 incidence rates and lesser severity in children and infants were attributed to lactoferrin present in breastmilk and lactoferrin-containing infant formulas widely used in this population [141]. Table 3 summarises the immune boosting functions and mechanisms of action of whey and casein.

#### 4.1.2. Casein

Bovine κ-Casein has been proven to have a direct inhibitory effect on the binding of viral particles via glycan residues against human rotavirus (HRV) [142]. This direct binding of viral particles results in 50–70% inhibition of viral activity against HRV, with the remaining 30–50% of uninhabitable activity hypothesised to be due to the fact there may be several key molecules involved in the cell entry process of viral attachment and replication [142]. In contrast, separate studies have shown that casein (the unmodified form) had no inhibitory effect on HIV-1 [143,144]. However, chemically modified casein inhibited HIV-1 via the direct binding of the HIV-1 gp 120 envelope glycoprotein and through direct binding of the CD4 cell receptor [145].

**Table 3:** Summary of immune mechanisms enhanced by milk-derived proteins.

Immune-Active Components	Immune-Boosting Functions	Mechanism	Reference
Whey/Lactoferrin	Antiviral	- Blocks viral attachment, replication, and production - Inhibits rdRp activity of SARS-CoV-2 - Inhibits reverse transcriptase, protease, integrase, and HIV-1 enzyme activity, inhibiting viral replication - ACE2 inhibitor	[132,134,135]
	Immunomodulator	- Promotes immune cell activation, differentiation, and proliferation - Induces type I interferon IFN-α/β - Promotes promoting CD4+ T cells into Th1 cells, stimulates neutrophil aggregation, activates phagocytosis, and increases activity of NK cells - Enhances antigen expression ability of B cells and regulates T cell function	[136,137,146,147]
	Anti-inflammatory	- Lowers IL-6 and TNF-α	[44]
Casein	Antiviral	- Inhibits viral binding in HRV via glycan residues - Some protease and integrase inhibitory activity - Potent inhibition of HIV-1 via direct binding of glycoprotein and CD4 cell receptor, inhibiting HIV-1 infection	[142,144,145]

### 4.2. Fermented Dairy Products

It is well documented that fermented foods can be used to support and boost immune responses in humans. For example, kefir, a fermented dairy product, has been noted for its antiviral and anti-inflammatory potential [148]. It can inhibit ACE levels and cholesterol metabolism, aid in wound healing, suppress tumour growth, alter the immune system to improve allergy symptoms, suppress viral activity via modulation of immune responses, and cause disruption of viral adhesion, as well as acting as an anti-inflammatory agent inhibiting proinflammatory cytokines like that of IL-1β, TNF-α, and IL-6 [148]. All of these are indicated in the low-grade inflammation seen within the elderly, obese, and immunocompromised populations, as well as being the key contributors to the cytokine storm of COVID-19 infection. Thus, kefir could be considered for its antiviral activity in the fight against COVID-19, largely through its ACE inhibitory abilities and its proinflammatory cytokine-reducing capabilities. Kefir is thought to exert this antiviral activity by direct probiotic–virus interaction and trapping, production of antiviral inhibitory metabolites, and/or via stimulation of the immune system for the development of bacteriocins, lactic acid, and hydrogen peroxide as antiviral agents [149]. Kefir modulates gut microbiota composition, regulates low-grade inflammation, controls intestinal permeability, and regulates gut homeostasis [150]; thus, it is a potentially powerful functional food for the elderly and IBD-immunocompromised and obese individuals whose gut immunity is compromised. Kefir improves serum zonulin levels, which are critical for the regulation of intestinal permeability and the modulation of tight junctions [151]. Furthermore, kefir could act against obesity by inhibiting enzymes related to the digestion of carbohydrates and lipids that result in less energy release [150].

Yogurt is a fermented milk product containing cultures of Lactobacillus bulgaricus and Streptococcus thermophilus [152]. Yogurt-derived peptides are known for their ACE inhibitory effects [153] and, therefore, may be effective in counteracting viral infection. Various in vitro and in vivo studies have shown that the bioactive peptides in yogurt have direct antiviral effects [153]. In addition to these antiviral effects, yogurt has been linked with improvements in gut health, reduced chronic inflammation by enhancing innate and adaptive immune responses, and improved intestinal barrier function [154]. Yogurt upregulates the expression of autophagy, tight junction proteins, and anti-microbial peptide-related genes, which all play a key role in maintaining a healthy gut barrier function through interaction with the intestinal epithelium [155]. Yogurt has inhibitory effects on colon cancer, restores gut homeostasis, and, therefore, prevents the development of and control of IBDs [156]. Decreases in TNF-α are associated with the consumption of LAB [157]. Therefore, yogurt is considered useful for the control of low-grade inflammation seen in the elderly, obese, and immunocompromised; for example, those suffering from type 2 diabetes [157]. Furthermore, yogurt also increases anti-inflammatory cytokine IL-10 while simultaneously reducing proinflammatory IL-17 and IL-12 [158], thus playing a key anti-inflammatory role crucial in the elderly, obese, and immunocompromised; in particular, those with IBDs.

Koumiss is a traditional fermented dairy product made from fermented mare’s milk originating in Mongolia [159,160]. Koumiss has been shown to have immunomodulatory capabilities by virtue of its ability to reduce TNF-α [161], a key player in the low-grade inflammation seen among the elderly, obese, and immunocompromised, as well as being a key contributor to the cytokine storm seen in COVID-19 infection. Koumiss has been shown to increase IFN-γ [161], and these IFN-γ secreting cells play a critical role in maintaining the gut barrier function. Furthermore, Koumiss is capable of inducing gut mucosal responses by enhancing the production of sIgA and therefore has effects on both the innate and adaptive immune responses [161]. SIgA prevents infection by inhibiting the attachment of bacteria and viruses within the gastrointestinal system [162].

Overall, fermented dairy products could be considered functional foods with the potential to protect against viral infection. These fermented foods can be highly beneficial for the elderly and obese and immunocompromised individuals through the modulation of gut microbiota composition and their overall antiviral abilities by virtue of their ACE inhibitory role, their direct viral inhibitory mechanisms, their gastrointestinal system maintenance, and their contribution to enhanced epithelial gut barrier function. Table 4 summarises the immune boosting functions and mechanisms of action of fermented food products, kefir, yoghurt, and Koumiss.

Furthermore, one food component of interest of late are fermentates. A fermentate generally refers to “a powdered preparation, derived from a fermented [food] product and which can contain the fermenting microorganisms, components of these microorganisms, culture supernatants, fermented substrates, and a range of metabolites and bioactive components” [163]. For example, an oral fermentation product known as EpiCor, derived from *Saccharomyces cerevisiae* (*S. cerevisiae*), showed the potential of enhancing the immune system to protect and aid in defense against cold/flu-like symptoms [164,165]. In these two 12-week randomized, double-blind, placebo-controlled trials, it was proven that this oral over-the-counter fermentate has the ability to reduce the incidence of cold and flu-like symptoms in both individuals with and without a history of influenza vaccination [165]. These studies show the potential of fermentates for the protection and prevention of viral infections and thus warrant further investigation into their potential uses against COVID-19 as well as other viral infections.

**Table 4:** Summary of immune mechanisms enhanced by fermented dairy products.

Immune-Active Components	Immune-Boosting Functions	Mechanism	Reference
Kefir	Antiviral	- Inhibits ACE levels and suppresses viral activity - Directs probiotic–virus interaction and trapping, production of antiviral inhibitory metabolites, and development of lactic acid and hydrogen peroxide as antiviral agents - Antioxidant	[148,149,153]
	Immunomodulator	- Enhances cholesterol metabolism, aids in wound healing, suppresses tumour growth, and improves allergy - Modulates gut microbiota composition, controls intestinal permeability, and regulates gut homeostasis - Improves zonulin levels and regulates intestinal permeability and modulation of tight junctions	[148,150,151]
	Anti-inflammatory	- Inhibits IL-1β, TNF-α, and IL-6	[148]
Yogurt	Antiviral	- ACE inhibitor	[166]
	Immunomodulator	- Antithrombotic - Improves gut health and intestinal barrier function - Upregulates expression of autophagy, tight junction proteins, and anti-microbial peptide-related genes for gut barrier health	[153,154,155]
	Anti-inflammatory	- Decrease TNF-α - Decreases IL-17 and IL-12 and increases IL-10	[157,158]
Koumiss	Antiviral	- Enhanced SigA production, inhibiting the attachment of viruses in the gastrointestinal tract	[161,162]
	Immunomodulator	- Maintains healthy gastric intestinal systems, regulates cholesterol and sugar levels, controls blood pressure, and produces important vitamins - Increases IFN-γ secreting cells to maintain gut barrier function	[161,167]
	Anti-inflammatory	- Decreases TNF-α	[161]

### 4.3. Plant-Derived Functional Foods

Plant-based functional foods are becoming increasingly more popular with the growing interest in vegetarian and vegan diets. Plant-based functional foods are derived from natural or unprocessed plant foods or may be derived from plant foods modified via biotechnological means [168,169]. Plants have been long known to have medicinal properties reducing the risk of developing a range of illnesses, including diabetes, cancer, cardiovascular disease, hyperlipidaemia, and hyperuricemia, by virtue of their immunomodulatory capabilities [170]. 

Virgin coconut oil (VCO) comes from the coconuts of coconut palm trees (Cocos nucifera) and is rich in nutrients, vitamins, and minerals, including vitamin E, palmitic acid, lauric acid, monolaurin, plant sterols, and bioactive compounds, including polyphenols, sterols, and tocopherols [171,172,173]. VCO has been noted for its anti-inflammatory, analgesic, [174], gut microbiota modulator [175], anti-stress, antioxidant [176], and antimicrobial activities [177]. Therefore, VCO is a potent functional food that possesses many desirable qualities that could aid in the boosting of immune fitness among the elderly, obese, and immunocompromised and could aid in the protection against viral infection and the promotion of gut homeostasis.

Recently, VCO has been highlighted as a potential antiviral functional food with the ability to lower CRP levels among suspect and probable COVID-19-infected patients, aiding in faster recovery from viral infection [171]. VCO has the ability to increase the phagocytotic activity of the innate immune macrophage [178] and has been shown to suppress and inhibit key inflammatory cytokines TNF-α, IFN-γ, IL-6, IL-8, and IL-5 [179]. Thus, it could be useful for the control of low-grade inflammation seen within the elderly and obese and immunocompromised individuals, as well as for the control of the cytokine storm observed in COVID-19 infection. VCO has been observed to have a positive effect on the adaptive immune response via the increased CD4^+^ T cell concentration, which is observed in HIV-positive individuals when supplementation with VCO is prescribed for 3 × 15 mL/day for 6 weeks [180], thus highlighting its importance as a functional food for the immunocompromised, including HIV-positive individuals. Similarly, VCO has been shown to increase CD4^+^ and CD8^+^ T cells in doxorubicin-induced immunosuppressed rats [181], showing its potential use for the elderly and immunocompromised and obese individuals, whose T cell levels are often compromised. Furthermore, more animal studies have shown the link between VCO consumption and increased adaptive immunity, where increased VCO consumption led to an increase in IgA in the spleen and Peyer’s patch cells of the small intestine [182].

Extra virgin olive oil (EVOO) is the least processed variety of olive oil, extracted from olives of the olive tree (Olea europaea) [183]. EVOO is rich in vitamins and minerals, including vitamin E, vitamin K, polyunsaturated fatty acids, oleic acid, and phenolic compounds like that of oleuropein and hydroxytyrosol [184,185,186]. In the US, a patent has been created that uses a naturally occurring secoiridoid glucoside oleuropein compound from Oleaceae plants in the treatment of viral diseases, such as hepatitis, mononucleosis, shingles, herpes, influenza, the common cold, and viral types causing leukemia [187]. Similarly, daily consumption of 50 g of EVOO in elderly HIV-positive individuals, without antiretroviral treatment, has been shown to improve lipid profiles and alpha diversity of intestinal microbiota, largely through the increase in *Bifidobacteriaceae* and *Gardnerella* species, and to decrease proinflammatory genera, such as *Dethiosulfovibrionaceae* [188]. In another study, high-sensitivity C reactive protein (CRP) concentrations were lowered in HIV-positive individuals receiving antiretroviral therapy after daily consumption of 50 mL EVOO [189]. Positive effects are seen on gut microbiota when EVOO is consumed via the reduction in pathogenic bacteria, the stimulation of beneficial bacteria, and the increase in the production of microbially produced short-chain fatty acids (SCFAs) to exert a wide range of anti-inflammatory effects [190]. EVOO influences intestinal mucosa and supports gut homeostasis by encouraging intestinal IgA production [191]. Polyphenolic compounds from EVOO have been linked to reduced T cell activation and proliferation as well as reduced proinflammatory cytokine secretion [192]. Other molecules in EVOO, such as oleuropein, reach the large intestine as unmodified compounds that the human colonic microbiota then catabolize to hydroxytyrosol; thus, there is much higher content of bioactive polyphenols present in the gut [186]. Therefore, EVOO could play a critical role in the control of viral infection seen in immunocompromised individuals like that of HIV sufferers, as well as the elderly and obese, where their viral immunity is already weakened. Table 5 summarises the immune boosting functions and mechanisms of action of plant-derived VCO, and EVOO.

### 4.4. Polyunsaturated Fatty Acids (PUFA)-Rich Foods

PUFAs act as substrates for proinflammatory and anti-inflammatory mediators, including prostaglandins, leukotrienes, thromboxanes, protectins, and resolvins [197], as well as for specialized pro-resolving lipid mediators (SPMs), which are critical chemical mediators needed for the stimulation of the resolution of inflammatory responses [198]. Omega-3 PUFA eicosapentaenoic acid (EPA) and docosahexaenoic acid (DHA) act as the substrate for SPM, while, in contrast, omega-6 PUFA arachidonic acid (AA) is the substrate for eicosanoids, including leukotrienes and prostaglandins, generated through the lipoxygenase and cyclooxygenase pathways [199]. Key sources of these omega-3 fatty acids are oily fish, such as salmon, mackerel, and trout, while omega-6 is found in meat, poultry, and eggs. A single lean fish meal, such as one serving of cod, could provide about 0.2 to 0.3 g of these omega-3 fatty acids, while a single oily fish meal, like one serving of salmon or mackerel, could provide 1.5 to 3.0 [200]. However, regardless of their wide availability, Western diets are often deficient in omega-3 PUFAs [129]. It is suggested that a dose of 60–90 mg of omega-3 PUFA could aid in the recovery of the gut microbiota and boost immunity [201].

Omega-3 PUFA has effects on both the innate and adaptive immune responses to aid in the tackling of invading viral particles. Omega-3 PUFAs upregulate the activation and improve the function of immune cells. For example, omega-3 PUFAs can induce cytokine and chemokine secretion and promote phagocytosis in macrophages [202]. Other effects of omega-3 PUFAs include increasing neutrophil function by enhancing migration, phagocytic capacity, and the production of reactive free radicals to kill microbes; promoting antigen presenting cells (APCs) that, in turn, activate T cells; inducing antibodies production in B cells; and boosting the first-line defense by activating dendritic cells, natural killer cells, mast cells, basophils, and eosinophils [197]. Long chain AA, EPA, and DHA have been shown to enhance epithelial barrier integrity as well as reduce IL-4-mediated permeability in gut [203]. A diet containing 18 g of fish oil/day for 12 weeks increased colonic concentrations of EPA and DHA while decreasing mucosal AA content in IBD [204]. Omega-3 PUFAs have the ability to modulate the gut microbiota [205] and have been shown to increase the abundance of several genera of gut microbes, including *Bifidobacterium* and *Roseburia* [206], of which a reduction in *Bifidobacterium* and *Lactobacillus* is implicated in many metabolic disorders and preserve a lean phenotype. Thus, omega-3 PUFAs are useful in the treatment and management of obesity [205,207]. *Bifidobacterium* and *Lactobacillus* have also been shown to improve clinical symptoms in IBDs [193]. These gut microbiota are critical for the continuous stimulation of resident macrophage within the intestine to release IL-10 for the promotion of Treg cells and the prevention of excessive Th17 cell activity [208]. Omega-3 PUFAs have been shown to increase triglyceride levels in patients with HIV, thus preventing lipid disorders, which could put the already at-risk individual at increased susceptibility to other diseases, including cardiovascular disease [209]. This increase in triglycerides through omega-3 supplementation could therefore be applied to the elderly population, too.

Omega-3-derived pro-resolving mediator protectin D1 has been associated with antiviral effects and inhibiting influenza viral replication in experimental models and thus warrants further investigation for its additive effect as a potential antiviral treatment for other lethal infections, such as COVID-19 [199]. Omega-3 PUFAs, including DHA-derived protectins and EPA-derived RvE1, have antiviral properties, with protectin D1 isomer (PDX) suppressing influenza virus replication through inhibition of the nuclear export of viral mRNA [210]. A link has been found between the supportive role of specialized pro-resolving mediators (SPM) in ARDS and acute lung injury [211]. Omega-3 supplementation has been shown to significantly improve ARDS patient status, including shorter duration of mechanical ventilation, shorter ICU stay, and significant decrease in ARDS mortality, and infectious complications remained unchanged [199]. These studies highlight the potential of omega-3 PUFAs as natural therapeutics for the treatment and prevention of viral infection, including influenza and COVID-19, and are thus of critical importance for the already at-risk elderly, obese, and immunocompromised individuals via their direct inhibition of viral replication.

It is hypothesised that by increasing omega-3 PUFAs and decreasing omega-6 PUFAs, one can skew the immune response in favour of the resolution of inflammation by favouring higher concentrations of resolvin/protectin rather than leukotriene/prostaglandin [199,212]. Omega-3 FAs are known to produce less pro-inflammatory cytokines; thus by increasing their intake as part of the diet, one could decrease viral entry, boost immune function, and even decrease the severity of disease in COVID-19 patients by virtue of altering the overdrive in immune response seen as the resultant cytokine storm [197]. Proinflammatory mediator gene activation is controlled by NF-kB, a transcription factor expressed in almost all cell types. Peroxisome proliferator-activated receptor (PPAR)-γ, an anti-inflammatory transcription factor, is activated by omega-3 PUFAs and leads to the inhibition of NF-kB activation; thus, the proinflammatory mediators cannot be transcribed [3,213]. NF-κB transcriptional activity and upstream cytoplasmic signaling events are downregulated by omega-3 FAs, EPA, and DHA [214]. Omega-3 FAs, EPA, and DHA downregulate the production of proinflammatory cytokines IL-1β, IL-6, and TNF-α associated with the aetiology of metabolic syndrome in THP-1-derived macrophages [214]. In particular, DHA has been linked to exerting an anti-inflammatory profile better than that observed from EPA [215]. Omega-3 PUFAs have been shown to reduce the ability of peripheral blood monocytes to produce TNF-α, IL-2, IL-1α, and IL-1β and to decrease mononuclear cell proliferation [216,217,218]. Thus, omega-3 PUFAs have the ability to decrease some of the key pro-inflammatory cytokines seen in the gut of the elderly, obese, and immunocompromised, which are exhibited as the chronic low-grade inflammation so detrimental to these at-risk individuals for increased susceptibility to viral infection. Omega-3 PUFAs are particularly potent in their ability to increase the IFN-γ /IL-4 ratio [219]. Stress-induced abnormalities in the intestine can be counteracted by DHA and EPA, reducing proinflammatory IFN-γ, TNF-α, IL-1β, and IL-6 while also increasing the expression of ZO-1, Z0-3 occluding, and E-cadherin [201,220]. Therefore, by increasing in particular DHA [221], one can inhibit the transcription of these proinflammatory genes by targeting their transcription factors and therefore aid in the modulation of the inflammatory process, thereby blocking the pathway and decreasing the cytokine storm seen in COVID-19 infection or decreasing the chronic low-grade inflammation seen in the elderly, obese, and immunocompromised. Mucous SIgA and serum IL-10 are increased at 60–90 mg doses of omega-3 PUFA [201], thus further exemplifying their potent anti-inflammatory effect. Further studies of the effect of omega-3 PUFA on dendritic cell function have demonstrated their role in increasing IL-10, suppressing IL-12, and enhancing the expression of CD40, CD80, CD86, and MHC II [215]. This suggests that omega-3 PUFAs could aid in the reduction of proinflammatory cytokines and the increase in anti-inflammatory IL-10 in the gut of the elderly, obese, and immunocompromised and potentially aid in the management of the chronic low-grade inflammation observed within these populations, as well as through inhibition of signaling pathways to control the hyperactivation of the inflammatory response.

It is thought that it is not only the COVID-19-induced cytokine storm that contributes to the overactive immune response that is so detrimental to the host individual, but also the so-called “eicosanoid storm”, which is characterized by increased levels of proinflammatory lipid mediators that are key to the development of severe infection [222]. Eicosanoids contribute to inflammation in a variety of ways, including the recruitment of inflammatory cells, vasodilation, and broncho- and vasoconstriction, as well as increased vascular permeability [199]. Studies have suggested that along with the cytokine storm, the eicosanoid storm of proinflammatory lipid mediators also contributes to the hyperinflammation that is so prevalent and detrimental to the COVID-19 infection [223]. Therefore, targeting of proinflammatory eicosanoid lipoxygenase and cyclooxygenase signaling pathways could provide a means of potential protective intervention against COVID-19 infection. Table 6 summarises the immune boosting functions and mechanisms of action of Omega-3, and Omega-6 PUFA.

### 4.5. Vitamin-D-Enriched Foods

Vitamin D is a crucial vitamin that helps regulate the amount of calcium and phosphate in the body in order to keep the bones and teeth strong and healthy, prevent the harmful effects of excess vitamin A, and prevent diseases like rickets and osteoporosis [225,226]. Furthermore, vitamin D is needed for muscle movement, nerve functioning, and for the immune system in helping to fight off invading bacteria and viruses [226]. The main source of vitamin D is from sunlight on our skin; however, it is also found naturally in foods, such as oily fish like salmon and sardines, as well as being sourced from eggs [225]. The vitamin D receptor is expressed on immune cells, including B cells, T cells, and APCs, which can synthesize the active vitamin D metabolite and therefore can potentially modulate both the innate and adaptive immune response, as deficiency in vitamin D is associated with increased susceptibility to infection [227]. It has been reported that poor nutrition and/or lack of sun exposure observed through low vitamin D levels contributes to severe disease and the progression of ARDS in some patients infected with COVID-19, while, similarly, low vitamin D levels in the active form of 1,25-dihydroxyvitamin D (1,25OHD) allow for proinflammatory molecules to trigger the subsequent development of ARDS in patients with COVID-19-associated pneumonia [228]. McCartney suggests that Irish adults should have an intake of 20–25 micrograms (800–1000 iu) of vitamin D per day for the duration of the COVID-19 pandemic [229], taken with food in order to achieve the critical 50 nanomoles per litre blood of vitamin D where immunity against COVID-19 can be enhanced [230]. These studies suggest that vitamin D is of critical importance to the elderly, obese, and immunocompromised, whose innate and adaptive immune responses are already weakened.

Vitamin D is predominantly present in the skin and thus functions in its active form, 1,25-dihydroxyvitamin D, along with vitamin D receptor (1,25OHD or VDR) to aid the immune system by maintaining tight junctions, gap junctions, and adherens junctions in the innate immune system [231]. Vitamin D supports the integrity of the epithelial barrier via the increased expression of VDR-associated intracellular junction proteins that constitute tight junctions between epithelial cells and include occludin, claudin, vinculin, ZO-1, and ZO-2 [232]. VDR is expressed in various tissues, including the skin, parathyroid gland, adipocyte, small intestines, and colon [233], and thus is widely expressed within the body, including within the gut; this means it could act as a therapeutic target where gut immunity is weakened. Vitamin D and VDR deficiency are associated with the pathogenesis of IBDs and is linked to elevated claudin-2 junction protein in inflammatory responses and therefore plays a critical role in intestinal barrier function [234]. VDR influences individual bacterial taxa, including *Parabacteroides*, where a much lower abundance of *Parabacteroides* are seen in UC and CD patients [235]. The downregulation of VDR or the inability to produce the active form of vitamin D is associated with a decrease in *Lactobacillus* in the gut and an increase in Proteobacteria [233], suggesting the influence of vitamin D on gut microbiota. Taken together, reductions in the levels of VDR and vitamin D are associated with dysfunctional intestinal integrity, intestinal barrier function, and gut microbiota composition; therefore, increased vitamin D consumption as a functional food component could aid in viral immunity and gut health for at-risk populations like the elderly and obese and immunocompromised individuals.

Active vitamin D suppresses Th1-mediated immune responses, inhibiting the production of inflammatory cytokines including IL-2 and IFN-γ while simultaneously promoting a Th2 response by producing anti-inflammatory cytokines IL-4 and IL-10 for indirect inhibition of the Th1 cells [231,236]. Furthermore, it induces Treg cells for the inhibition of the inflammatory process for the overall inhibition of a viral attack [231]. Deficiency in vitamin D negatively impacts Treg differentiation and weakens its function, thus leading to the triggering of autoimmune diseases, including IBDs [237]. Correcting vitamin D deficiency has been associated with suppressed CD26 adhesion molecules used for COVID-19 cell adhesion and invasion, as well as being linked to the ability to attenuate IFN-γ and IL-6 inflammatory responses, both of which are highly correlated with critically ill, ventilated COVID-19 patients [229] and within the elderly, obese, and immunocompromised.

Taken together, these mechanisms of antiviral activity via the suppression of proinflammatory markers could potentially be applied to the chronic low-grade inflammation seen in the elderly, obese, and immunocompromised, or for the cytokine storm that occurs during COVID-19 infection. These mechanisms work via the targeting of cell surface adhesion molecules for the suppression and/or inhibition of the otherwise dangerously proinflammatory state leading to chronic disease persistence or viral infection. Table 7 summarises the immune boosting functions and mechanisms of action of vitamin-D enriched foods.

### 4.6. Zinc-Enriched Foods

Zinc is a key micronutrient involved in the maintenance of a healthy immune system, directly affecting aspects of the innate and adaptive immune responses [239]. Zinc can be found in food sources including oyster, red meat, and poultry, as well as in smaller amounts in beans, nuts, and whole grains [240]. Zinc deficiency occurs frequently in the elderly and the obese, as well as those with chronic diseases, such IBDs [241,242,243]. Zinc supplementation has been shown to have protective effects against viruses like the common cold and to result in fewer infectious incidents, including pneumonia in the elderly [50]. Zinc deficiency is responsible for 16% of all deep respiratory infections worldwide [244], which suggests a link between deficiency in zinc and the risk of infection and severe prognosis of COVID-19. This suggests a possible role for supplementation as a treatment or preventative antiviral measure [245].

Zinc enhances mucociliary clearance of viruses like the coronaviruses, removing the viral particle and reducing the risk of secondary infections; it is also essential for preserving tissue barrier integrity and important in protecting against viral entry into a host [245]. Zinc deficiency has been associated with reduced first responder cellular chemotaxis and phagocytosis, while supplementation has proven to enhance this [239]. Zinc has the potential to increase the cytotoxic activity of natural killer cells (NK), which are capable of attacking the cells that have abnormal or unusual proteins in the plasma, by infecting the cells and causing the microorganisms within the cells to be released and destroyed through phagocytosis by neutrophils and macrophages [246]. Furthermore, zinc deficiency is linked to altered MHCI recognition by NK cells and thus influences NK lytic abilities [239]. MHCI recognition is needed to allow NK cells to function to their best ability in order to kill the invading virus. Macrophage function becomes reduced when an individual is zinc deficient and when oxidative burst becomes impaired, while, in addition, neutrophil granulocytes cause reduced chemotactic activity and decreased numbers [247]. Zinc deficiency is associated with the pathogenesis of CD due to poor zinc absorption in the gastrointestinal lumen of the small intestine [248]. Zinc deficiency is associated with decreases in transepithelial resistance and alterations in the tight and adherens junctions, including ZO-1, occluding, *β*-catenin, and E-cadherin, leading to the disruption of membrane barrier integrity and the subsequent infiltration of neutrophils [249]. Furthermore, zinc-dependent alterations in gene expression by pneumocytes also affect viral entry: whereby zinc binding the ACE2 active centre becomes essential for enzymatic activity, zinc homeostasis might affect ACE2 expression, which is regulated by Sirt-1 and which zinc decreases; thus, this might decrease ACE2 expression and subsequent viral entry into cells [245].

In addition to this, zinc directly inhibits viral replication for many viral infections, including influenza, HIV, *papillomaviridae*, *picornaviridae*, *Herpesviridae*, *metapneumovirus*, and coronavirus (SARS-CoV); thus, due to their similarity, it is estimated that this is likely to also be true for SARS-CoV-2 [243,245]. The mechanism by which it is thought to do so is by preventing fusion with the host membrane, decreasing viral polymerase function, impairing protein translation and processing, blocking particle release, and destabilising the viral envelope [243]. Long-term zinc supplementation at nutritional levels delays immunological failure, decreases diarrhea, and decreases rates of opportunistic infection over time in HIV-positive patients [250]. It is thought that zinc can inhibit HIV reverse transcriptase presumably via the competitive displacement of one or more Mg^2+^ ions bound to the reverse transcriptase, with zinc promoting the formation of a highly stable, slowly progressing reverse transcriptase complex [251]. Low-dose supplementation of zinc in combination with zinc ionophores, such as pyrithione and hionkitol, can decrease RNA synthesis in influenza, poliovirus, picornavirus, equine arteritis virus, and SARS-CoV by directly inhibiting RNA-dependant RNA polymerase (rdRp) [245,252].

Zinc deficiency influences the adaptive immune system, causing T cell lymphopenia [253]. Too high or too low levels of zinc have been linked to the inhibition of nicotinamide adenine dinucleotide phosphate (NADPH) oxidases, which enable the destruction of invading pathogens [254]. Thus, it is important to strike a balance in the levels of zinc in the body to reach an optimal zinc homeostasis to avoid immunosuppression via supplying zinc in either excess or deficient quantities. Zinc deficiency is characterised by an increase in proinflammatory cytokines like IL-1β, IL-6, and TNF-α, all of which are elevated during COVID-19 infection [239], and chronic low-grade inflammation within the gut of the elderly, obese, and immunocompromised. Similarly, zinc deficiency results in increased IL-8 and thus plays a critical role in gut inflammation [249]. Zinc acts as an anti-inflammatory to maintain immune tolerance via the induction of Treg cell development and mitigates the development of proinflammatory Th17 and Th9 cells, thus limiting the inflammatory response and controlling low-grade inflammation seen in the elderly, obese, and immunocompromised [246]. Zinc, when supplemented with antiretroviral therapy in HIV patients, has been shown to increase CD4^+^ T cell counts as opposed to antiretroviral treatment alone [243]. Managing proinflammatory cytokines is key to the prevention of the cytokine storm and chronic low-grade inflammation seen in the elderly, obese, and immunocompromised. Zinc possesses antiviral and anti-inflammatory activity through its ability to inhibit NF-κB signaling and the modulation of regulatory T-cell functions and thus can limit the cytokine storm in COVID-19 and chronic low-grade inflammation [255].

It has been observed that there is a clear link between zinc deficiency and viral infections, including HIV and COVID-19 [256,257]. Patients in the at-risk group for contracting COVID-19 and who are at risk of a poorer prognosis of COVID-19 have been highly interlinked to lower zinc levels [256,257]. Such groups include individuals with chronic obstructive pulmonary disorder (COPD), bronchial asthma cardiovascular diseases, autoimmune diseases like UC and CD, and kidney diseases, dialysis patients, as well as those with comorbidities, such as obesity, diabetes, cancer, atherosclerosis, liver cirrhosis, immunosuppression, and known liver damage [256,257]. Thus, it is important to consider the possible role that zinc homeostasis has in the prevention and protection from contracting COVID-19 and other viral infections, as it is clear that it plays a critical role in antiviral immunity, where its deficiency is already seen to be strongly correlated with poorer clinical outcomes and is therefore of critical importance to the already at-risk elderly, obese, and immunocompromised populations. Table 8 summarises the immune boosting functions and mechanisms of action of zinc enriched foods.

### 4.7. Selenium-Enriched Foods

Selenium is a ubiquitous element to sulfur that is found in nature and can be sourced organically from food [259]. Selenium constitutes 25 selenoproteins that play critical roles in reproduction, thyroid hormone metabolism, DNA synthesis, protein folding, mitochondrial health, and, most importantly, protection from oxidative damage and defense against viral infection [260,261,262]. Selenium deficiency is a risk factor for several chronic diseases associated with oxidative stress and inflammation, including IBDs [263], as well as being associated with obesity [264]. Selenium functions by virtue of its selenocysteine-active centre [261]. Rich sources of selenium include eggs, fish, corns like wheat, maize, and rice, chicken liver, garlic, onions, broccoli, yeast bran, coconut fruits, Brazil nuts, and seafood, and it is an essential component of all living organisms [265]. Selenium deficiency is reported to affect 500 million to 1 billion people worldwide, mainly due to inadequate dietary intake [261].

Selenium regulates the intestinal microflora, with increased gut microbiota diversity observed with increased dietary selenium, which in turn affects the gut microflora, influencing selenium bioavailability and selenoprotein expression [266,267,268]. Increases in proinflammatory taxa, including *Turicibacter and Dorea*, are associated with IBD [269,270]. With moderate selenium consumption, microbiota including *Turicibacter and Dorea* can be regulated and intestinal damage can be improved [267,271]. Selenium deficiency affects the killing ability of NK cells [271].

Deficiency in selenium leads to increased viral pathogenesis via oxidative stress and redox signaling, which ultimately affects cell proliferation, apoptosis, and cytokine expression [272,273]. Oxidative stress is a result of viral infections causing a disruption to the equilibrium between reactive oxygen species (ROS) and their scavenging systems, thus causing an imbalance between ROS and the cellular antioxidant defense system [274]. Viral infections result in oxidative stress, enhancing the replication and accumulation of mutations in the viral RNA genome, which ultimately leads to increased virulence and damage to the host via this amplification loop [274]. Deficiency in selenium has been associated with mutations in the viral genome that result in highly virulent forms of the viral particle, as well as being linked with increased susceptibility and pathogenicity of viral infections [274]. Selenoproteins are essential for an effective immune response to infections [261], largely through the critical selenoproteins, glutathione peroxidase and thioredoxin reductases, that provide antiviral defense by virtue of their redox signaling and homeostatic activities [262]. Selenium’s antiviral activity is largely controlled by antioxidant factors, including glutathione peroxidase (GPXs) regulation by selenocysteine [272]. Furthermore, selenium has been shown to demonstrate an inhibitory effect on HIV via the antioxidative effects of GPX and other selenoproteins, with low selenium levels being correlated to HIV-infected individuals and further disease progression [275]. Similarly, selenium deficiency is seen in patients with hepatitis B and C viruses, and increases in selenium would help see better treatment response [276]. Selenite acts as an oxidant, which has important implications for selenium’s antiviral properties, in that selenite reacts readily with sulfhydryl groups in the active site of viral protein disulphide isomerase (PDI), converting them to inactive disulphide; thus, the viral hydrophobic spike loses its ability to undergo the exchange reaction with disulphide groups of the cell membrane proteins and therefore renders the virus unable to enter the healthy cell cytoplasm, preventing viral entry into the cell [277,278].

Selenium status has been found to positively correlate with the survival of patients with COVID-19 compared with non-survivors, while overall selenium levels are lower in patients with COVID-19 than their healthy control counterparts [261]. This suggests the importance of adequate selenium levels in the prevention of COVID-19 and could further suggest its relevance as an antiviral for other viral infections. These viral mechanisms contribute to the oxidative stress associated with many RNA viral infections, the increased viral replication and hence increased mutation rate, and the higher pathogenicity or even higher mortality seen in selenium-deficient patients with COVID-19; thus, there is a clear association being reported between cure rates for COVID-19 and selenium status, as observed through the examination of city-based population selenium statuses of different Chinese cities [279]. Similarly, these findings have been clinically confirmed in Germany, where serum selenium levels were shown to be highly correlated with COVID-19 outcomes in hospitalised patients; 65% of those who died had low selenium compared to only 39% of those who survived, and very low selenium levels were present in 44.4% of patients. Most importantly, the lowest selenium levels were strongly associated with mortality, thus highlighting the importance of selenium in the defense and protection against severe clinical outcomes in COVID-19 patients [280].

Furthermore, selenium is a well-known NF-*k*B inhibitor and thus plays a critical role in reducing viral-induced apoptosis; it could also influence the mitigation of the cytokine storm in COVID-19 infection [281,282] and chronic low-grade inflammation seen in the gut of the elderly, obese, and immunocompromised by virtue of the interruption of the signaling pathway responsible for the chronic proinflammatory state. NF-*k*B is the central mediator of immune and inflammatory responses critically responsible for the proinflammatory cytokine production involved in the life-threatening cytokine storm [282] and chronic low-grade inflammation within the gut. Supplementing at-risk groups, including the elderly, obese, and immunocompromised, with 200 mcg selenium supplementation daily for three weeks, followed by a maintenance dose of less than or equal to 200 mcg μg for the duration of the active circulation of COVID-19, as well as the documentation of serum selenium levels in COVID-19-hospitalised patients for the systemic addition of selenium upon hospitalisation at the earliest stage possible, could aid in the management of the cytokine storm [282].

Selenium deficiency is linked to increases in proinflammatory cytokines IL-6, IL-8, IFN-γ, and TNF-α, while decreases in anti-inflammatory cytokines IL-2, IL-10, IL-17, IL-1β, IFN-α, and IFN-β have been observed in many tissues, including the gastrointestinal tract [283,284]. Selenium supplementation increases the polarization of macrophages from the M1 to M2 phenotype, favouring inflammatory resolution, playing a critical role in IBDs [285]. This suggests selenium’s role as an anti-inflammatory capable of managing the chronic low-grade inflammation seen in the gut of the elderly, obese, and immunocompromised through regulation of the proinflammatory immune response via cytokine production and their signaling pathways.

Selenium plays a key role in the proliferation and differentiation of CD4+ Th cells [271]. Increases in selenium result in increased Treg cell differentiation from naïve CD4+ T cells through TCR stimulation [271]. Therefore, increased selenium may play a role in managing the chronic inflammation of IBDs and low-grade inflammation seen in the gut of the elderly, obese, and immunocompromised by virtue of its regulatory role in T cell differentiation. Table 9 summarises the immune boosting functions and mechanisms of action of selenium enriched foods.

## 5. Conclusions and Outlook

**Figure 4:**
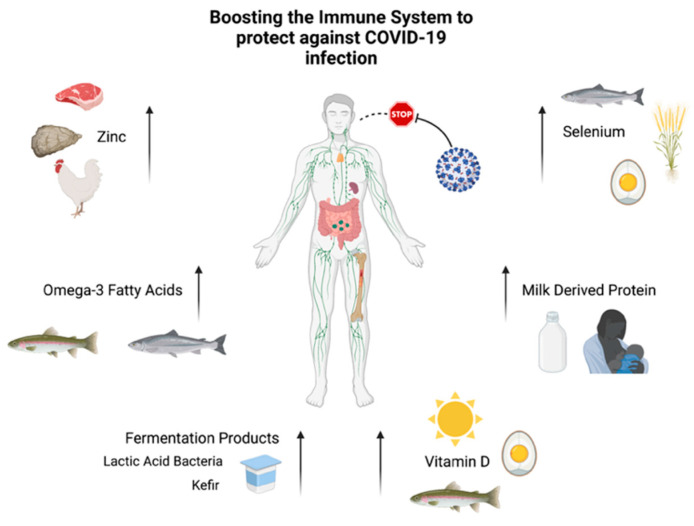
Summary of functional foods capable of boosting the immune system and inhibiting COVID-19 infection. Functional foods with properties capable of inhibiting viral infection and their key food sources. Arrows indicate increasing food component consumption to aid in preventing COVID-19 infection. Created using BioRender.com.

In summary, the COVID-19 pandemic has led to a focus on potential treatment and prevention methods to control, limit, and halt the spread of the virus, which causes an array of symptoms and illnesses, including mild to severe symptoms, such as ARDS, multiple organ failure, and, ultimately, death. An examination of the added risk of other comorbidities and age is an important challenge in the fight against COVID-19. In this review, we examined the potential of functional foods as natural sources of immune-boosting reinforcements at a time when antiviral vaccines are under strain due to rapid mutation and high turnaround for boosters due to their low 6-month immunity. Various studies have started to reveal that milk proteins, dairy fermentation products, and food products containing PUFAs, vitamin D, selenium, and Zinc may be used in the development of functional foods with the potential to combat not only COVID-19 but also other viral infections via their immune-modulating capabilities (Figure 4). This review, therefore, stresses the importance of supporting immune fitness by means of healthy eating and increased functional food intake, particularly for at-risk individuals, including the elderly, the obese, and those who are already immunocompromised.

## Figures and Tables

**Table 1:** Functional food market size in Ireland, Eastern Europe, Western Europe, and the world.

Year	2016	2017	2018	2019	2020	2021	2022	2023	2024	2025	2026
Ireland	494.97	510.65	519.51	531.83	554.24	584.79	585.71	591.82	617.89	646.61	679
Eastern Europe	4142	4727.30	4967.70	4999	4921	5211.80	5530.50	5745.60	5977.00	6234.30	6498.10
Western Europe	20,689.50	20,695.50	21,568.80	20,661.60	21,537.40	23,032.30	23,526.50	23,906.90	24,444.30	25,130.70	25,803.50
USA	31,902.50	31,708.50	31,007.50	30,956.90	32,053.90	31,512.30	32,940.60	34,148.30	35,239.50	36,210.90	37,164.30
World	159,040.10	164,365.30	168,157.20	168,477.90	168,919.00	177,395.00	184,464.70	192,846.20	201,417.00	201,355.60	219,467.80

Market values from 2016 to 2021 in USD million. Predicted market values for 2022–2026 in USD million. Data obtained from Euromonitor International, a market research provider (2022).

**Table 2:** Definitions of the term “functional food” and their originating regions.

Country	Definition	Reference
EU	A product which is shown in a satisfactory manner that, in addition to adequate nutritional effects, induces beneficial effects on one or more target functions of the organism, significantly improving the health status and welfare or reducing the risk of disease.	[124]
USA	Foods that, by virtue of the presence of physiologically active components, provide a health benefit beyond basic nutrition	[125]
Canada	Similar in appearance to conventional food, consumed as part of the usual diet, with demonstrated physiological benefits, and/or to reduce the risk of chronic disease beyond basic nutritional functions	[126]
Japan	Known as Foods for Specified Health Use, these are foods composed of functional ingredients that affect the structure and/or function of the body and are used to maintain or regulate specific health conditions, such as gastrointestinal health, blood pressure, and blood cholesterol levels	[127]

**Table 5:** Summary of immune mechanisms enhanced by plant-derived functional foods.

Immune-Active Components	Immune-Boosting Functions	Mechanism	Reference
Virgin Coconut Oil	Antiviral	- Faster recovery from COVID-19 - Disrupts the virus envelope, inhibits pathogen maturation, prevents assembly and budding of viral progeny, prevents pathogens from directly binding to the host cells, and inhibits production of viral particles - Antioxidant	[171,173,193]
	Immunomodulator	- Increases phagocytosis of innate macrophage - Anti-ulcerative, reduces gastric juice, reduces total acid output, reduces ulcer scoring, and increases gastric wall mucous secretion - Increases CD4+ T cell concentration in HIV patients - Increases CD4+ and CD8+ T cells - Increases IgA in spleen and Peyer’s patch cells in small intestine	[178,180,181,182,193]
	Anti-inflammatory	- Lowers CRP levels - Inhibits TNF-α, IFN-γ, IL-6, IL-8, and IL-5	[171,179]
Extra Virgin Olive Oil	Antiviral	- Antioxidant	[194].
	Immunomodulator	- Improves lipid profiles and alpha diversity of intestinal microbiota - Reduces pathogenic gut microbiota and increases beneficial bacteria - Influences intestinal mucosa, supports gut homeostasis, and encourages intestinal IgA production - Reduces T cell activation and proliferation	[188,190,191,192]
	Anti-inflammatory	- Increases production of SCFA in gut - Lowers CRP concentrations in HIV patients - Reduces proinflammatory cytokine secretion - Reduces IL-6, TNF-α, metalloprotease secretion, COX-2, and α-smooth-actin levels - Inhibits IL-8, IL-6, NF-kB activation, and iNOS induction	[189,190,192,195,196]

**Table 6:** Summary of immune mechanisms enhanced by polyunsaturated fatty acids (PUFA)-rich foods.

Immune-Active Components	Immune-Boosting Functions	Mechanism	Reference
Omega-3 PUFA e.g., EPA, DHA Omega-6 PUFA e.g., AA	Antiviral	- Pro-resolving mediator protectin D1 inhibits influenza virus replication - PDX suppresses influenza virus replication by inhibition of nuclear export of viral mRNA	[199,210]
	Immunomodulator	- Upregulates the activation and improves the function of macrophage to promote cytokine and chemokine secretion and improve phagocytosis - Enhances neutrophil migration and production of free radicals, enhances T cell production through APCs, improves B cell function to produce more antibodies - Enhances CD40, CD80, CD86, and MHCII - Improves first-line cellular defense, producing more dendritic cells, NK cells, mast cells, basophils, and eosinophils - Enhances epithelial barrier integrity - Modulates the gut microbiota, increasing microbes including Bifidobacterium, Roseburia, and Lactobacillus - Increases triglyceride levels in patients with HIV - Improvements in ARDS patients through SPM - Increases mucous SIgA	[197,199,201,202,203,205,206,209,211,215]
	Anti-inflammatory	- Reduces IL-4-mediated permeability in the intestine - Activates PPAR-γ transcription factor, inhibits NF-kB activation - Reduces production of TNF-α, IL-2, IL-6, IL-1α, and IL-1β and decreases mononuclear cell proliferation - Suppresses IL-12, increases IL-10 - Increases IFN-γ/IL-4 ratio - Reduces IFN-γ and increases expression of ZO-1, Z0–3, and E-cadherin - Reduces omega-6 eicosanoids and aids in the resolution of eicosanoid storm	[3,199,201,203,214,215,216,217,218,219,220,224]

**Table 7:** Summary of immune mechanisms enhanced by vitamin-D-enriched foods.

Immune-Active Components	Immune-Boosting Functions	Mechanism	Reference
1,25-dihydroxyvitamin D and vitamin D receptor (1,25OHD or VDR)	Antiviral	- Suppresses CD26 adhesion molecules, inhibits COVID-19 cell adhesion and invasion	[229],
	Immunomodulator	- Maintains tight, gap, and adherens junctions - Supports integrity of epithelial barrier and increases expression of VDR-associated intracellular junction proteins, including occludin, claudin, vinculin, ZO-1, and ZO-2 - Improves gut barrier function - Influences gut microbiota - Induces B cell proliferation and the secretion of IgE and IgM, enables formation of memory B cells and B cell apoptosis promotion	[231,232,233,234,238]
	Anti-inflammatory	- Suppresses Th1-mediated immune responses (inhibits IL-2 and IFN-γ), promotes Th2 response (produces IL-4 and IL-10) - Induces Treg cells - Attenuates IL-6	[229,231,236]

**Table 8:** Summary of immune mechanisms enhanced by zinc-enriched foods.

Immune-Active Components	Immune-Boosting Functions	Mechanism	Reference
Zn^2+^	Antiviral	- Enhances mucociliary clearance of viruses, removes the viral particle, reduces risk of secondary infections, preserves tissue barrier integrity to prevent viral entry - Inhibits ACE2 - Inhibits viral fusion with host membrane, decreases viral polymerase function, impairs protein translation and processing, blocks particle release, and destabilises the viral envelope - Inhibits HIV reverse transcriptase - Decreases RNA synthesis of viruses by direct inhibition of rdRp	[243,245,251,252]
	Immunomodulator	- Increases first responder cellular chemotaxis and phagocytosis - Increases cytotoxic activity of NK cells - Influences NK lytic abilities via MHCI recognition by NK cells - Regulates transepithelial resistance and tight and adherens junctions, including ZO-1, occluding, β-catenin, and E-cadherin, thus influencing membrane barrier integrity - Modulates NADPH oxidases - Stimulates production of IgG - Increases premature and immature B cells and affects antibody production	[239,246,247,249,254,258]
	Anti-inflammatory	- Reduces IL-1β, IL-6, and TNF-α - Decreases IL-8 - Induces Treg cell development, mitigates Th17 and Th9 - Inhibits NF-κB signaling and modulates Treg cell function - Increases CD4+ T cell counts in HIV patients	[239,243,246,249,255]

**Table 9:** Summary of immune mechanisms enhanced by selenium-rich foods.

Immune-Active Components	Immune-Boosting Functions	Mechanism	Reference
Selenite Selenoproteins	Antiviral	- Resists viral genome mutations, prevents development of highly virulent forms of viral particles, decreases susceptibility and pathogenicity of viral infections - Antiviral defense through redox signaling and homeostatic activity - Antioxidant - Inhibits HIV and slows HIV disease progression - Improves treatment response in HBV and HBC patients - Prevents viral entry to cell via interaction of sulfhydryl in active site of viral PDI	[262,272,274,275,276,277,278]
	Immunomodulator	- Regulates intestinal microflora, increases gut microbiota diversity, influences selenium bioavailability and selenoproteins’ expression - Modulates microbiota, including Turicibacter and Dorea, and improves intestinal damage - Improves NK killing ability	[266,267,268,271]
	Anti-inflammatory	- Inhibits NF-kB - Decreases IL-6, IL-8, IFN-γ, and TNF-α - Increases IL-2, IL-10, IL-17, IL-1β, IFN-α, and IFN-β - Increases polarisation of M1 to M2 phenotype - Enhances CD4+ Th proliferation and differentiation - Increases Treg cell differentiation	[271,281,282,283,284,285]

## Data Availability

No new data were created or analysed in this study. Data sharing is not applicable to this article.
